# Investigation of the Relationship between Permanent Deformation and Dynamic Modulus Performance for Bearing-Layer Asphalt Mixture

**DOI:** 10.3390/ma16196409

**Published:** 2023-09-26

**Authors:** Weidong Ji, Yunrui Meng, Yunlong Shang, Xiwei Zhou, Huining Xu

**Affiliations:** 1 School of Transportation Science and Engineering, Harbin Institute of Technology, Harbin 150001, China; wedoji@163.com (W.J.); yunruimeng2@gmail.com (Y.M.); 20b932029@stu.hit.edu.cn (X.Z.); 2 Heilongjiang Transportation Investment Group Co., Harbin 150028, China; myrstu1@163.com

**Keywords:** asphalt mixture, bearing layer, permanent deformation, dynamic modulus, control standard

## Abstract

Of major concern is the lack of correlation between the material design and structural function of asphalt pavement in China. The objective of this paper is to identify the layer in asphalt pavement where permanent deformation occurs most seriously and to propose a control index for that layer’s asphalt mixture. The permanent deformation of each layer was determined through the utilization of thickness measurements obtained from field cores. The results indicate that the reduction in thickness is more significant in the driving lane than in the ridge band and shoulder. This phenomenon can be attributed to the intensified densification and shearing deformation that arise from the combined impacts of recurrent axle loads and high temperatures. Compared to surface and base layers, the bearing layer is the primary area of concern for permanent deformation in asphalt pavement. Therefore, it is imperative to incorporate the ability of bearing-layer asphalt mixture to withstand permanent deformation as a crucial design parameter. The dynamic modulus of the bearing-layer asphalt mixture is significantly influenced by the type of asphalt, gradation, and asphalt content, compared to other design parameters. Based on the relationship established between dynamic modulus and dynamic stability, with creep rate as the intermediate term, a control standard was proposed to evaluate the permanent deformation of the bearing-layer asphalt mixture. This study can provide reasonable and effective guidance for prolonging pavement life and improving pavement performance.

## 1. Introduction

Asphalt pavement has become the most important form of road paving in China due to its good driving comfort, excellent road performance, and convenient maintenance characteristics [1,2]. During the service life of asphalt pavement, various types of distress commonly occur. Therefore, understanding the material parameters and pavement conditions is crucial for durability and performance assessments. The composition, layer thickness, and material properties (e.g., density, stiffness, elasticity, and thermal conductivity) of pavement material are parameters that need to be taken into consideration [3]. To determine material parameters, certain algorithms have been created which are based on the mechanical features of pavement. For example, the multi-level inverse algorithm is designed to simultaneously identify the stiffness and thickness of pavement models [4]. Utilizing visual and non-destructive testing methods, recent research has advanced surface quality assessment [5,6]. Garbowski et al. [7] used three-dimensional laser scanning of a road to identify pavement deterioration type and its quantity. De Blasiis et al. [8] developed a program for processing 3D point cloud data to identify and quantify a few types of pavement distress. By utilizing these technologies, we can assess and study the existing pavement to direct the pavement design.

Designing the asphalt mixtures is a key factor in ensuring the quality of asphalt pavement. Asphalt pavement is constructed layer by layer, and each layer has its own functional emphasis and stress characteristics [9]. Specifically, for a pavement structure with three layers of asphalt pavement, the surface layer ensures driving comfort, the bearing layer mainly resists permanent deformation, and the base layer is more critical in resisting fatigue [10,11]. With the increase in pavement service life and traffic loading, permanent deformation has become one of the main sources of the distress of asphalt pavement. Permanent deformation has a significant impact on the performance of asphalt pavement, particularly as the permanent deformations trap water and cause hydroplaning [12,13]. Moreover, significant permanent deformation can lead to major structural failures [14]. Hence, the permanent deformation not only reduces the service life, but it may also affect the safety of highway users.

It is well known that the resistance to permanent deformation is closely related to the pavement materials and structures. According to the research conducted by Xu et al. [10], the primary source of vertical deformation in asphalt pavement is the shearing deformation of its bearing layer. From the perspective of the mechanical response of asphalt pavement under traffic load, the bearing layer is in the high-shear-stress area. Li et al. [11] discovered that the bearing layer of the asphalt mixture became the primary compressed layer as permanent deformation formed, based on their measurements of the thickness of field cores. The study by Zhao et al. [15] delved into the factors that impact the permanent deformation of asphalt pavement. The researchers analyzed 180 field cores and determined that road age and equivalent single-axle loads were the primary determinants of permanent deformation of the pavement structure. Through the implementation of the Hamburg wheel tracking test, Zhu et al. [16] appraised the permanent deformation of the bearing layer of an existing highway. Evidence from the literature suggests that the bearing layer of asphalt can suffer permanent deformation, and this should be taken into account when designing asphalt mixtures. At present, the Marshall method is used in China for asphalt mixture design. The volume parameter is typically adopted as the primary design index, which utilizes the dynamic stability as the verification parameter for permanent deformation. It creates a disconnect between the material and structural design of asphalt pavement and makes a significant difference in the anti-permanent-deformation requirements of the bearing-layer asphalt mixture. Therefore, it is necessary to study the design index for bearing-layer asphalt mixture based on permanent deformation.

Various endeavors have been undertaken to establish testing methodologies, and evaluation parameters that can effectively measure the permanent deformation of asphalt mixtures, including the dynamic modulus test, dynamic creep test, repeated-load permanent deformation test, Hamburg wheel tracking test, etc. [17,18,19,20]. The relevant research results of the Long-Term Pavement Research Program in the United States show that the dynamic modulus has a good correlation with the rut depth in the field, which can be used to evaluate the permanent deformation of asphalt mixture [21]. The permanent deformation of six asphalt mixtures were investigated by Zhang et al. [22], who discovered that the dynamic creep is strongly correlated with the outcomes of dynamic modulus and repeated-load permanent deformation tests. Chaturabong et al. [23] observed that dry Hamburg wheel tracking was strongly correlated with the deformation in the second stage of the dynamic creep test, and that it can simulate field behavior very well. Witczak et al. conducted a comprehensive study on the dynamic modulus. The test results show that there is a high correlation between permanent deformation and the dynamic modulus of asphalt pavement [24]. By utilizing these methods and assessment criteria, the capacity of asphalt mixture to withstand permanent deformation can be more accurately determined. However, the complexity of the development of permanent deformation is due to many influential factors, such as binder type, asphalt content, mixture type, load level, temperature, etc. [10,17,20,24,25]. Therefore, it is crucial to choose the appropriate method to evaluate the permanent deformation in the design of asphalt mixtures.

According to the aforementioned studies, the asphalt mixtures’ design is crucial in ensuring the performance of asphalt pavements. The purpose of this study is to choose a suitable design parameter for permanent deformation of bearing-layer asphalt mixture and establish a control standard. Intuitively, the layer’s thickness can reflect the depth and distribution of the permanent deformation in each layer. We obtained core samples from the selected highway sections, calculated the contribution rate of permanent deformation for each layer, and identified the main layer and potential causes of the permanent deformation which had occurred in the asphalt pavement. An analysis was conducted using the local sensitivity method to evaluate the impact of asphalt type, gradation type, and asphalt content on the dynamic modulus, compressive strength, resilient modulus, shear strength, and splitting strength of bearing-layer asphalt mixtures. The significant performance parameter was chosen to establish a relationship with dynamic stability. Based on this, a control standard was suggested for the evaluation of the permanent deformation, one that connects the material design and the structural design of the bearing-layer asphalt mixture. This paper provides reasonable and effective guidance for prolonging the service life and improving the performance of asphalt pavement.

## 2. Investigation of Permanent Deformation of Asphalt Pavement in Northeast China

### 2.1. Road Section Information

**Figure 1:**
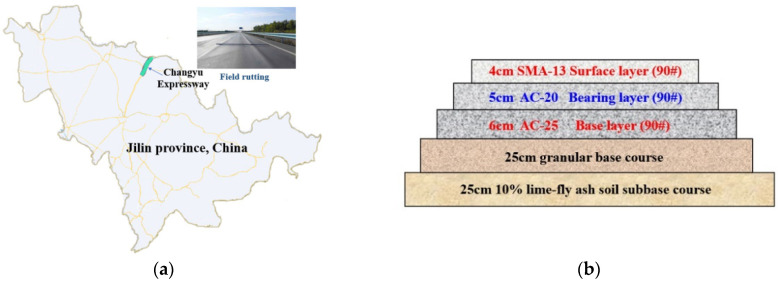
Information as to the selected expressway: (**a**) location and photograph of Changyu Expressway, (**b**) pavement structures.

The investigation of permanent deformation of asphalt pavement is the basis for analyzing the causes of the permanent deformation dysfunction and the structural function of the pavement. The Changyu Expressway in Jilin Province was selected (Figure 1a). It is one of the primary trunk highways, and has been in service for 12 years. The selected expressway is 143.5 km long and 26.0 m wide. A schematic diagram of the pavement’s structure is shown in Figure 1b.

**Figure 2:**
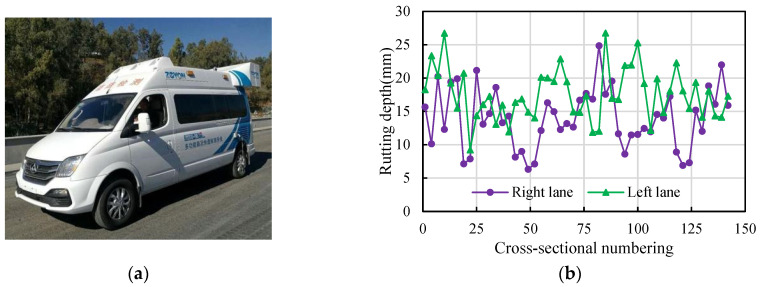
Field investigation: (**a**) ZOYON-RTM detection vehicle, (**b**) results of permanent deformation depth detection.

The ZOYON-RTM intelligent road comprehensive detection vehicle (Figure 2a) was used to identify the permanent deformation state of Changyu Expressway. The detection system uses high-frequency, high-precision line-scan imaging 3D data acquisition technology to automatically obtain permanent deformation depth. The detection speed was 35 km/h and the temperature in the field was 30 °C. The permanent deformation depth in the typical pavement segment selected is shown in Figure 2b. The permanent deformation depth on the Changyu Expressway ranges from 6.31 mm to 26.75 mm, and the average value is 15.79 mm.

### 2.2. Field Cores Information

**Figure 3:**
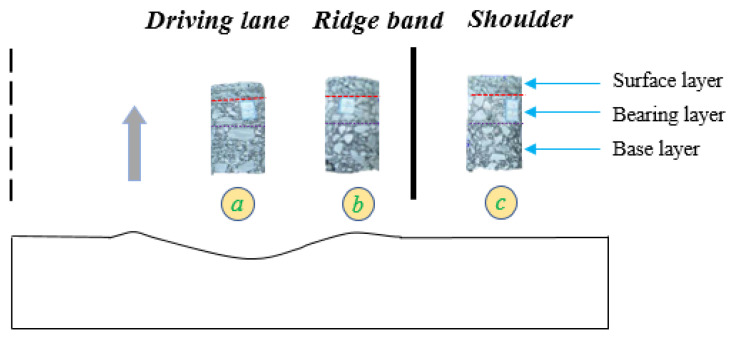
Schematic diagram for the selection of field cores.

The aim was to enhance our understanding of the root causes of permanent deformation and to determine the contribution of each asphalt pavement layer to the permanent deformation. To achieve this goal, a set of full-depth core samples (with a diameter of 100 mm) were extracted. These samples were extracted from wheel paths in the same cross-section in the driving lane, the ridge band between wheel paths and shoulder, and the pavement shoulder, respectively. The layout scheme of field cores in a section is illustrated in Figure 3. One core (a) was drilled on a wheel path, one core (b) was drilled on the ridge band, and one core (c) was drilled on the road shoulder. Through examination of the changes in thickness within the field cores, the layer in the asphalt pavement where permanent deformation significantly occurs can be identified. Evidence from the literature shows that permanent deformation is closely related to the pavement materials and structures [11,15]. Hence, the design index for permanent deformation should be considered in the material design of the asphalt mixture located in that layer.

## 3. Materials and Methods

### 3.1. Materials and Sample Preparation

The types of asphalt utilized in this study include 30# and 90# ordinary petroleum asphalt, as well as rubber-modified asphalt. The basic properties were tested following the Standard Test Methods of Bitumen and Bituminous Mixtures for Highway Engineering (JTG E20-2011) [26]. The results are presented in Table 1. The coarse and fine aggregates of the asphalt mixture were made of andesite, which is obtained from Heilongjiang Province. The mineral filler was crushed limestone. The properties of coarse aggregates and mineral filler are given in Table 2 and Table 3, respectively. The technical indicators of the aggregate and mineral filler meet the requirements of the Technical Specification for Construction of Highway Asphalt Pavements (JTG/F40-2004) [27].

**Figure 4:**
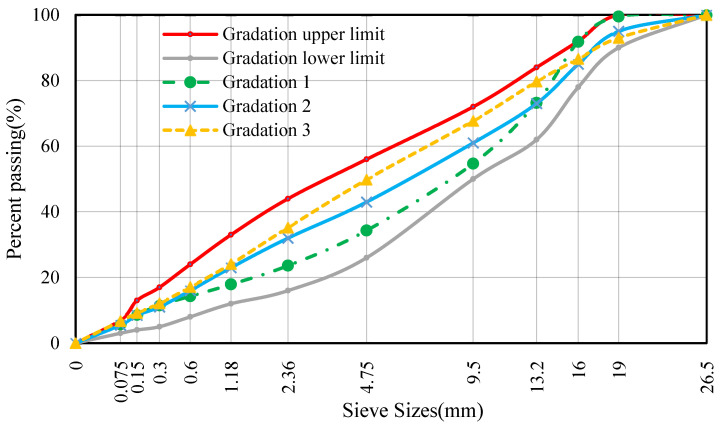
Aggregate gradation curves.

AC-20 mixture is commonly used in China for the construction of the bearing layer [14,16]. To clarify the influence of aggregate gradation on asphalt mixture performance, a comparative analysis was conducted on three AC-20 gradation options. The gradation composition of these asphalt mixtures is shown in Figure 4.

### 3.2. Test Methods

The purpose of choosing a property test index was to direct the asphalt mixture design towards resisting permanent deformation. As mentioned above, dynamic modulus is an important parameter used to measure the elastic properties of asphalt mixture. Uniaxial compressive strength can reflect the resistance of asphalt mixture to compressive deformation. Shear strength can represent the shear capacity of asphalt mixture. Splitting strength can measure the tensile capacity of asphalt mixture. Flow number and creep rate can reflect the ability of asphalt mixture to resist high-temperature deformation. The specific test methods are as follows.

#### 3.2.1. Dynamic Modulus Test

A dynamic modulus test was carried out according to the Chinese specification JTG E20-2011. The specimens had heights of 150 mm and diameters of 100 mm. The test temperature was 25 °C. The frequencies were 25 Hz, 10 Hz, 5 Hz, 1 Hz, 0.5 Hz, and 0.1 Hz. The dynamic modulus has a certain stress dependence. When the dynamic loading frequency is constant, the dynamic modulus increases with an increase in stress [28]. Dynamic modulus was calculated as follows:

(1) $$|E^{*}|=\frac{\sigma_{0}}{\varepsilon_{0}}$$

where $\left|E^{*}\right|$ is the dynamic modulus (MPa), $\sigma_{0}$ is the axial stress amplitude value (MPa), and $\varepsilon_{0}$ is the axial strain amplitude value (mm/mm).

#### 3.2.2. Uniaxial Compression Test

Uniaxial compression strength was obtained following the Chinese specification JTG E20-2011. The specimens had heights of 100 mm and diameters of 100 mm and had been manufactured on a shear gyratory compactor. The test temperature was 15 °C. The loading rate was 2 mm/min. There is a positive correlation between unconfined compressive strength and the capability to withstand permanent deformation. Uniaxial compression strength was obtained as follows:

(2) $$R_{c}=\frac{P}{A}$$

where $R_{c}$ is the uniaxial compression strength (MPa), *P* is the peak load at sample failure (N), and *A* is the cross-sectional area of the sample (${mm}^{2}$).

#### 3.2.3. Uniaxial Penetration Test

Shear strength was evaluated in accordance with the Specifications for Design of Highway Asphalt Pavement (JTG D50-2017) [29]. The specimens had heights of 100 mm and diameters of 100 mm and had been manufactured on a shear gyratory compactor. The test temperature was 60 °C. The loading rate was 1 mm/min. The greater the shear strength, the better the shear-deformation resistance of the asphalt mixture. Shear strength was computed as follows:

(3) $$\tau_{0}=\frac{fP}{A}$$

where $\tau_{0}$ is the shear strength (MPa), *f* is the shear stress coefficient, *P* is the peak load at sample failure (N), and *A* is the cross-sectional area of the penetration mold (${mm}^{2}$).

#### 3.2.4. Splitting Test

Splitting strength was tested referring to the Chinese specification JTG E20-2011. The specimens were prepared by the standard Marshall method. The test temperature was 25 °C. The loading rate was 50 mm/min. The greater the splitting strength, the better the deformation resistance of the asphalt mixture. Splitting strength was calculated as follows:

(4) $$R_{T}=\frac{0.006287P_{T}}{h}$$

where $R_{T}$ is the splitting strength (MPa), $P_{T}$ is the failure load (N), and *h* is the specimen height (mm).

#### 3.2.5. Dynamic Creep Test

The dynamic creep test is used to measure the flow number and creep rate of an asphalt mixture. To eliminate the effect on the Marshall sample during the initial loading stage, a preload of 0.002 MPa was applied for 10 min before loading. Then, a compressive stress of 0.05 MPa was applied. The loading waveform was a half sine wave, and the loading period was 1 s, including 0.1 s of loading and 0.9 s of unloading. The third stage of the test is characterized by the emergence of cracks in the specimen or a discernible deviation in the test curve, events which serve as the termination criteria [30]. Creep rate was obtained as follows:

(5) $$\varepsilon_{s}=\frac{\varepsilon_{2}-\varepsilon_{1}}{{(t}_{2}-t_{1})\sigma_{0}}$$

where $\varepsilon_{s}$ is the creep rate (1/s/MPa), $\sigma_{0}$ is the flexural–tensile stress (MPa), and $t_{1},t_{2},\varepsilon_{1}$,$\varepsilon_{2}$ are the starting times and ending times of the stable period and the corresponding creep-strain values.

In this study, three types of asphalt (30# and 90# ordinary road petroleum asphalt, and rubber-modified asphalt), three AC-20 gradations (1, 2, and 3) and five asphalt content levels (3.9%, 4.2%, 4.5%, 4.8%, and 5.1%) were selected to investigate the influence on design parameters of the asphalt mixture. Three parallel sets of specimens were prepared for all tests that were carried out by the Universal Testing Machine-250 (IPC Global, Wantirna South, Australia), and the value of each test index was obtained by the average value of the three parallel specimens. Before testing, all the samples were stored in a temperature-controlled chamber at a specified temperature for 4~5 h.

## 4. Results and Discussion

### 4.1. Thickness Changes in Each Layer of the Field Cores

**Figure 5:**
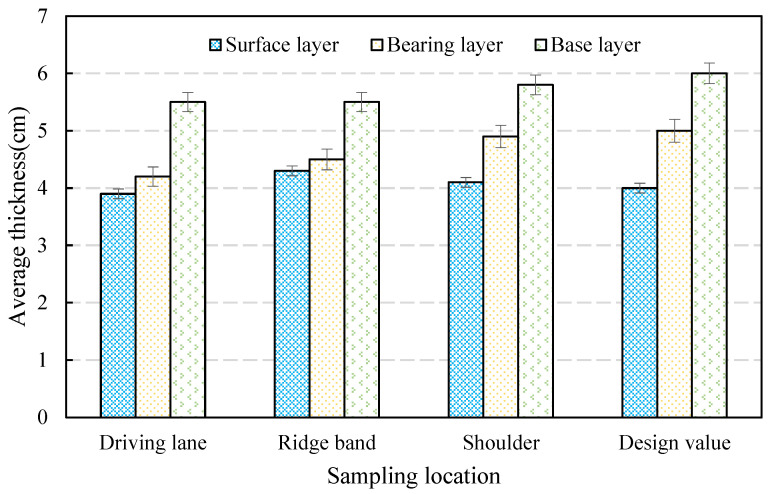
The thickness changes of each asphalt pavement layer.

As mentioned in Section 2.2, in order to improve the ability of the asphalt pavement to resist permanent deformation, it is feasible to identify the layer where permanent deformation mainly occurs and optimize the design indices of this layer. Intuitively, the layer’s thickness can reflect the depth and distribution of the permanent deformation in each layer. According to previous studies [10,11], the total pavement deformation in a typical pavement structure occurred solely in the three asphalt mixture layers above the granular base course. Hence, our analysis was limited to measuring the thicknesses of the three distinct layers to identify the layer where permanent deformation occurs most significantly. We selected five cross-sections with a permanent deformation depth of 15 mm and measured the thicknesses of the core specimens. The testing results for the thicknesses of each layer are shown in Figure 5.

From analysis of Figure 5, it can be determined that the reduction in thickness is more significant in the driving lane than in the ridge band and shoulder. This phenomenon can be attributed to the intensified densification and shearing deformation that arise from the combined impacts of recurrent axle loads and high temperatures [10,16]. The deformation of the asphalt mixture layer is the predominant cause of rutting. Specifically, for the driving lane, a minor alteration of 1.1 mm was observed in the thickness of the surface layer, which decreased from 40.0 to 38.9 mm. As a result of a thickness alteration in the bearing layer, the measurement decreased from 50.0 to 42.0 mm, which was accompanied by a variation of 8.0 mm. This change accounts for 53.3% of the total rutting depth. The thickness of the base layer changed from 60.0 to 55.1 mm, with a variation of 4.9 mm, accounting for approximately 32.7% of the whole rutting depth. In general, the bearing layer experiences the most severe rutting deformation and accounts for the majority of the deformation. Based on the available data, it appears that the bearing layer is the primary area of concern for rutting deformation in asphalt pavement. Therefore, it is imperative to incorporate the ability of bearing-layer asphalt mixture to withstand permanent deformation as a crucial design parameter.

### 4.2. Design Parameters of Asphalt Mixture in the Bearing Layer

At present, the relationship between asphalt mixture design and actual pavement performance is empirical. The Marshall method used in China is used for mixture design. Its design indices are volume parameters (porosity, asphalt saturation, mineral aggregate gap probability, etc.) and two mechanical indices (Marshall stability and flow value), which omits consideration of the structural role of the asphalt mixture in the bearing layer in the design index. This leads to a disconnection between the material design and the structural design of the bearing-layer asphalt mixture. In this study, we aim to obtain a mechanical index related to pavement performance in order to guide the material design of the bearing layer. This design index is determined by taking into account the dynamic modulus, compressive strength, resilient modulus, shear strength, and splitting strength.

#### 4.2.1. Analysis of Factors Influencing Design Parameter

**Figure 6:**
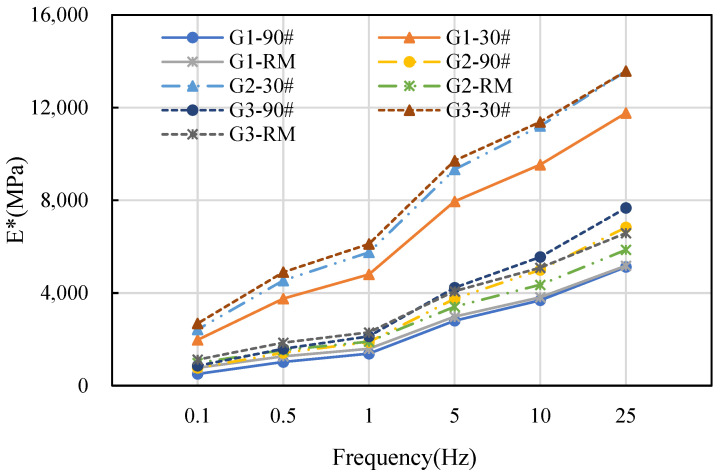
Dynamic modulus curves at different frequencies, with asphalt content of 4.5%. (Notes: G1-90# means asphalt mixture with gradation one and 90# asphalt; G means gradation; 30# means 30# asphalt; RM means rubber-modified asphalt).

**Figure 7:**
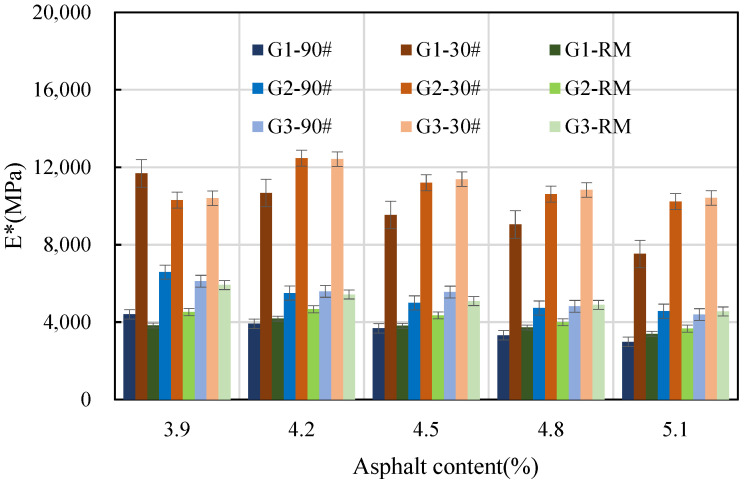
Dynamic modulus at different asphalt contents with frequency of 10 Hz.

A comprehensive analysis of the mechanical indices of asphalt mixture was conducted when the gradation type, asphalt type, and asphalt contents were altered. Among them, the modulus of the material is an important parameter of the pavement structure [31]. Taking the dynamic modulus as an example, the results are shown in Figure 6 and Figure 7.

Figure 6 presents the dynamic modulus curves at different frequencies, with an asphalt content of 4.5%. The dynamic modulus exhibits an increase as the frequency increases. The reason for this is that when the frequency is modified, the load will not be compressed to its full extent, and the “unload” will not bounce back entirely, resulting in energy buildup. As the frequency increases, the energy levels rise, and the strain decreases when subjected to constant stress. Therefore, the dynamic modulus will gradually increase.

Furthermore, the types of gradation and asphalt also have an impact on the dynamic modulus of asphalt mixtures. When comparing within gradation one, it was found that the dynamic modulus of 30# asphalt mixture was nearly double that of the 90# asphalt mixture with the same frequency and 4.5% asphalt content. However, the difference between the dynamic modulus of 90# asphalt mixture and rubber asphalt mixture was minimal. Similar results were obtained for asphalt mixtures when comparing within gradations two and three. For asphalt mixtures with the same type of asphalt but different gradations, the dynamic modulus has little difference under the condition of constant loading frequency and 4.5% asphalt content. Comparing the dynamic modulus values of gradations one, two, and three, it can be observed that gradation three has the highest modulus, followed by gradation two and then gradation one. It can be inferred that the dynamic modulus of asphalt mixtures is impacted by the type of gradation and asphalt.

A loading frequency of 10 Hz and a loading time of 0.016 s have been found to produce an effect on asphalt pavement comparable to that of a speed of 60–65 km/h. Therefore, the loading rate of 10 Hz is equivalent to the effects of the actual road speed [32]. To streamline the analysis process, the dynamic modulus at 10 Hz was adopted as the standard for analysis in subsequent tests. The dynamic modulus values at different asphalt contents with a frequency of 10 Hz are shown in Figure 7.

As shown in Figure 7, within a certain range of asphalt content, the dynamic modulus of 90# asphalt mixture shows a monotonically decreasing trend with an increase in asphalt content. For 30# asphalt and rubber-modified asphalt mixtures, the dynamic modulus initially increased and then decreased with the increase in asphalt content. And the inflection point occurs at 4.2% asphalt content. However, the dynamic modulus of the rubber-modified asphalt mixture with gradation three decreased monotonously with an increase in asphalt content. The amount of asphalt has the greatest impact on the dynamic modulus of the 90# asphalt mixture, while the dynamic modulus of the rubber asphalt mixture is the least affected by changes in the amount of asphalt. The dynamic modulus of the 30# asphalt mixture falls between these two extremes. This shows that the content of asphalt has a great influence on the dynamic modulus of asphalt mixtures.

The test results of other design indices are shown in Table 4, including compressive strength, resilient modulus, shear strength, and splitting strength. The index value of each experimental condition was obtained from the mean value of three parallel experiments. It can be seen that these indices are significantly affected by asphalt type and gradation type. For asphalt type, under the condition of a certain gradation and asphalt content, the compressive strength, resilient modulus, and splitting strength of 30# asphalt mixture are the highest, followed by those of rubber-modified asphalt mixture, and 90# asphalt mixture returns the lowest values. The shear strength of rubber-modified asphalt mixture is the highest, followed by 30# asphalt mixture, and 90# asphalt mixture returns the lowest values. Under the condition of a certain asphalt type and asphalt content, comparing these indices of gradations one, two, and three, it can be observed that gradation three has the highest values, followed by gradation two and then gradation one. As to the content level of asphalt, with an increase in asphalt content under the set condition of a certain gradation and asphalt type, these mechanical indices initially increase and then decrease. The peak points are mainly at 4.2% and 4.5% asphalt content, findings which are basically consistent with the conclusion of the dynamic modulus. This also indicates that the optimal amounts of asphalt for asphalt mixtures with different gradations and types are 4.2% and 4.5%. The above analysis shows that gradation, asphalt type, and asphalt content have effects on the mechanical index of an asphalt mixture.

#### 4.2.2. Sensitivity Analysis

The sensitivity of each design index to asphalt type, gradation type, and asphalt content was analyzed by the single-factor analysis of variance method. The calculation results are shown in Table 5. The results of the single-factor analysis of variance were subjected to a 95% confidence level. A variable can be deemed to have a significant impact on the design index value of the asphalt mixture if its significance index *p* value is less than 0.05 [19].

According to the results of the single-factor variance analysis in Table 5, the dynamic modulus is sensitive to a change of asphalt content within the same gradation and asphalt type. The splitting strength is not sensitive to changes in asphalt content. The compressive strength, resilient modulus, and shear strength are partially sensitive to changes in asphalt content, but partially insensitive. Therefore, it is reasonable to take the dynamic modulus as the design index for asphalt mixtures. Meanwhile, in the case of a limited sample size of asphalt mixture variations, the type of asphalt had the most significant influence on the dynamic modulus. This means that the type of asphalt has an important effect on the permanent deformation of asphalt mixtures. Compared with modifying the gradation type or the asphalt content, changing the type of asphalt is more helpful in improving the resistance to the permanent deformation of the asphalt mixture.

### 4.3. Control Standard for Asphalt Mixture in the Bearing Layer

The connection between permanent deformation and dynamic modulus has been poorly investigated in existing studies. Through extensive research on the correlation between dynamic creep and dynamic modulus, the connections between permanent deformation, dynamic modulus, and dynamic creep have been established by these findings. As a result, the dynamic modulus is proposed as the design index for bearing-layer asphalt mixture in accordance with different permanent-deformation control standards.

#### 4.3.1. Analysis of Factors Influencing Dynamic Creep

**Figure 8:**
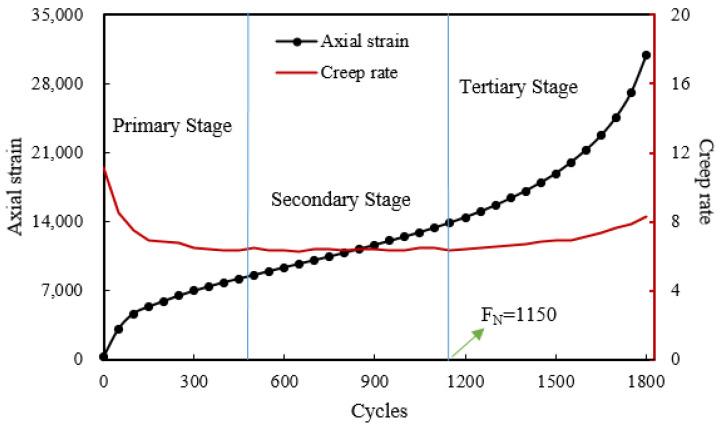
Three stages of creep development.

Empirical evidence suggests that the dynamic creep test has a very strong correlation with permanent deformation depth and a high capability to estimate the potential of permanent deformation [33]. The dynamic creep test has been used to judge and predict permanent deformation, and to investigate the creep law and extent of deformation of asphalt mixtures under repeated loads [34,35]. According to existing research results, creep development of asphalt mixtures has three distinct strain stages [36]: (1) primary stage, in which the strain rate decreases; (2) secondary stage, in which the strain rate is constant; and (3) tertiary stage, in which the strain rate increases. The loading cycle corresponding to the deformation curve upon reaching the third stage is defined as the flow number (F_N_). During the stable period, the rate at which strain increases per unit of time under a unit of stress is known as the creep rate. The flow number and creep rate are commonly used as indicators to evaluate the stability of asphalt mixtures at high temperatures [37]. Figure 8 shows an example of a creep curve.

**Figure 9:**
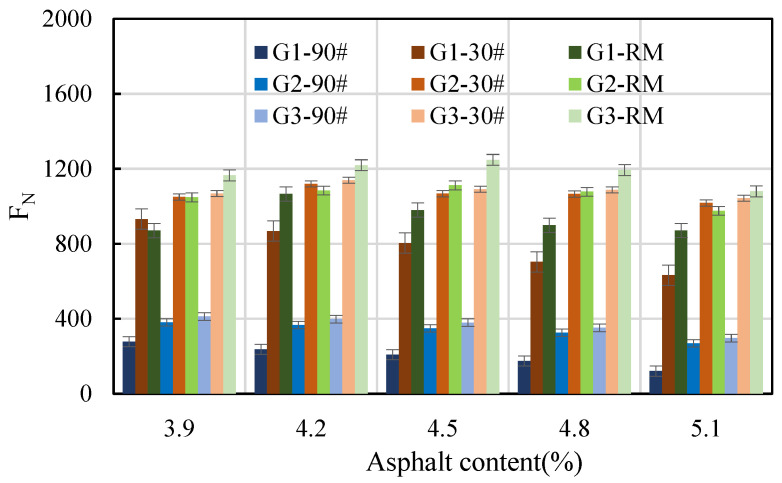
Flow number at different asphalt contents.

**Figure 10:**
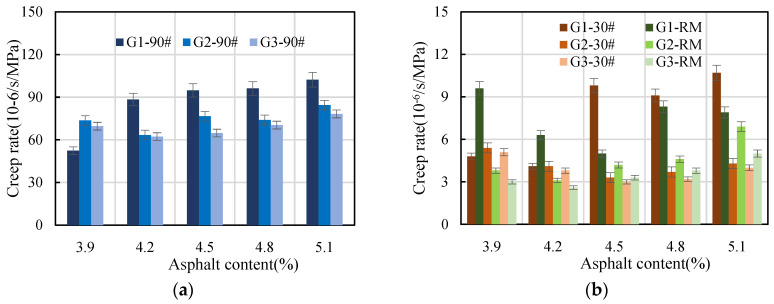
Creep rate at different asphalt contents: (**a**) 90#, (**b**) 30# and RM.

In this study, the impact of dynamic creep was examined using three types of asphalt (30# and 90# ordinary petroleum asphalt, and rubber-modified asphalt), five asphalt content levels (3.9%, 4.2%, 4.5%, 4.8%, and 5.1%), and three AC-20 gradations (one, two, and three). The flow number and creep rate at different asphalt contents are shown in Figure 9 and Figure 10, respectively.

The flow number is used to describe the permanent-deformation behavior of an asphalt mixture under loading stress with time. A higher flow number indicates a greater resistance to permanent deformation for the asphalt mixture. As seen in Figure 9, for asphalt mixtures with the same gradation and asphalt content, the flow number of the 30# ordinary asphalt and rubber-modified asphalt mixture were not significantly different, but they were significantly higher than that of the 90# asphalt mixture. This indicates that 90# asphalt mixture has poor high-temperature resistance. For the asphalt mixture with the same asphalt content, the flow number of gradation 1 was the smallest, followed by that of gradation two, and the flow number of gradation three was the largest. Within a certain range of asphalt content, the flow number of the 90# asphalt mixture decreased monotonically with an increase in asphalt content. For the 30# asphalt, both alone and in rubber-modified asphalt mixtures, the flow number of the 30# asphalt mixture with gradation one decreased monotonically with an increase in asphalt content. However, the flow numbers of the other mixtures first increased and then decreased with an increase in asphalt content. The inflection points appeared at the asphalt contents of 4.2% and 4.5%. The results indicate that the flow number of asphalt mixture is significantly affected by the asphalt type, gradation type, and asphalt content.

Creep rate serves as an indicator of the permanent-deformation performance of asphalt mixture under loading stress with time. The smaller the creep rate, the stronger the permanent-deformation resistance of the asphalt mixture. As shown in Figure 10, for asphalt mixtures with the same gradation and asphalt content, the creep rate of 30# ordinary asphalt and rubber-modified asphalt mixture showed little difference, but they were significantly lower than that of the 90# asphalt mixture. This also indicates that the permanent deformation resistance of 90# asphalt mixture is poor. For asphalt mixtures of the same type of asphalt and asphalt content level, the general rule was that the creep rate of gradation one was the highest, followed by that of gradation two, and the creep rate of gradation three was the lowest. Within a certain range of asphalt content, the creep rate of asphalt mixture varied depending on the type of asphalt. Except for the 90# asphalt mixture with gradation one, whose creep rate increased monotonically with an increase in asphalt content, the creep rate of other asphalt mixtures first decreased and then increased with the increase in asphalt content. The inflection point occurred when the asphalt content was between 4.2% and 4.5%. The results show that the creep rate of asphalt mixture is also greatly affected by asphalt type, gradation type, and asphalt content.

#### 4.3.2. Relationship between Dynamic Modulus and Permanent Deformation

**Figure 11:**
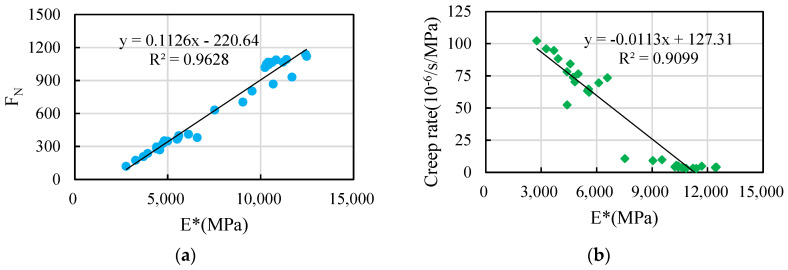
Relationship between dynamic modulus and creep parameters with 30# and 90#: (**a**) flow number, (**b**) creep rate.

**Figure 12:**
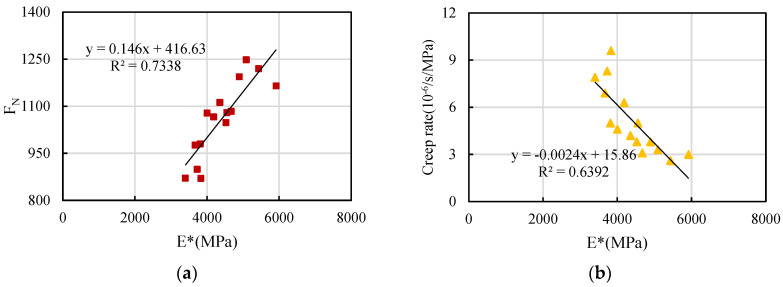
Relationship between dynamic modulus and creep parameters with RM: (**a**) flow number, (**b**) creep rate.

As evident from the outcomes mentioned earlier, the creep rate and flow number exhibit clear trends with variations in asphalt content, and effectively differentiate the permanent deformation of asphalt mixture. Based on the experimental results for dynamic modulus, the correlation between creep parameters and dynamic modulus indices is analyzed below. Among these values, the dynamic modulus at 10 Hz is selected, because the loading rate of 10 Hz is equivalent to the effects of the actual road speed [32]. The relationship between the dynamic modulus and creep parameters of ordinary petroleum asphalt mixtures and rubber-modified asphalt mixtures, respectively, is shown in Figure 11 and Figure 12.

Figure 11a conveys that the flow number of ordinary petroleum asphalt mixture has a strong correlation with the dynamic modulus. The flow number increases linearly with the increase in dynamic modulus. The correlation coefficient between them is 0.9628. As shown in Figure 11b, it is evident that the creep rate exhibited by ordinary petroleum asphalt mixture is positively correlated with the dynamic modulus. The creep rate decreases linearly with an increase in dynamic modulus. The correlation coefficient between them is 0.9099.

It can be seen from Figure 12a that the flow number of rubber-modified asphalt mixture has a certain correlation with the dynamic modulus. The flow number increases with an increase in dynamic modulus. The correlation coefficient between them is 0.7338. The correlation between the creep rate of rubber-modified asphalt mixture and the dynamic modulus is evident in Figure 12b. The creep rate decreases with an increase in dynamic modulus. The correlation coefficient between them is 0.6392.

As evidenced by Figure 11 and Figure 12, the relationship between creep parameters and dynamic modulus is more pronounced for ordinary asphalt mixture in comparison to rubber-modified asphalt mixture. The correlation between flow number and dynamic modulus is higher than that between creep rate and dynamic modulus. Therefore, the flow number is chosen as the assessment criterion for creep testing in order to carry out standardized investigations on dynamic modulus parameters.

In the literature, the analysis of dynamic creep test results and rutting test results performed by Qi Feng indicates that there is a good relationship between the flow number and permanent deformation [38]. The relationship is as follows (the correlation coefficient *R*^2^ is 0.927):

(6) $$DS=2.8099\cdot F_{N}+535.6$$

According to the analysis in Figure 12a, when the test temperature is 25 °C and the loading frequency is 10 Hz, the relationship between the flow number and the dynamic modulus is as follows (the correlation coefficient *R*^2^ is 0.963):

(7) $$F_{N}=0.1126\cdot E^{*}-220.6$$

The connection between permanent deformation and dynamic modulus can be derived by merging Formulas (6) and (7).

(8) $$DS=0.3146\cdot E^{*}-84.3$$

By utilizing the flow numbers and dynamic modulus test results at different frequencies determined at 25 °C, the correlation between permanent deformation and dynamic modulus can be determined. The results are summarized in Table 6.

The results presented in Table 6 demonstrate that when frequency and temperature remain constant, an increase in dynamic stability leads to a higher dynamic modulus. By referring to Table 6 or utilizing an interpolation method, one can acquire the dynamic modulus values of asphalt mixtures according to various permanent-deformation control standards. For example, when the permanent deformation of asphalt mixture is required to be ≥2400 times/mm, it can be found by using Table 6 that E* ≥ 7851 MPa is obtained when the loading frequency is 10 Hz. This is basically consistent with the requirements of the Chinese specification JTG D50-2017.

## 5. Conclusions

This study focused on an investigation of the relationship between permanent deformation and dynamic modulus performance in bearing-layer asphalt mixtures. The following conclusions were drawn.

Intuitively, the layer’s thickness can reflect the depth and distribution of the permanent deformation in each layer. Through field investigation and coring, it was determined that the reduction in thickness is more significant in the driving lane than in the ridge band and shoulder. This phenomenon can be attributed to the intensified densification and shearing deformation that arise from the combined impacts of recurrent axle loads and high temperatures. Compared to surface and base layers, the bearing layer is the primary area of concern for rutting deformation in asphalt pavement. Therefore, it is necessary to strengthen the evaluation of the high-temperature performance of bearing-layer asphalt mixture.

By sensitively analyzing the results, it can be determined that the dynamic modulus of the bearing-layer asphalt mixture is significantly influenced by the type of asphalt, gradation type, and asphalt content, compared to other design parameters. The relationships between dynamic modulus, flow number, and permanent deformation were combined to establish the control standard for a bearing-layer asphalt mixture. The dynamic modulus should not be less than 7851 MPa when the permanent deformation of asphalt mixture is required to be ≥2400 times/mm. This research can provide a mechanical index associated with pavement performance, one which can be utilized to guide material design and consequently extend the lifespan of the pavement, as well as enhance its performance.

## Figures and Tables

**Table 1:** Base asphalt properties.

Properties		Asphalt (30#)	Asphalt (90#)	Rubber-Modified Asphalt	Methods
Ductility (cm)	15 °C	56	>100	/	T0604-2011
	5 °C	/	/	41	
Penetration degree at 25 °C (0.1 mm)		29.0	83.7	65	T0605-2011
Softening point (°C)		73.2	51.4	65.6	T0606-2011
Dynamic viscosity at 60 °C (Pa·s)		742	187	13014	T0620-2011
Viscosity at 135 °C (Pa·s)		0.67	1.56	8.65	T0625-2011

**Table 2:** Coarse-aggregate properties.

Technical Indices	Results	Criteria	Methods
Crush value (%)	5	≤28	T0316-2005
Content of acicular and flaky shape particles (%)	8.6	≤15	T0312-2005
Losses of the Los Angeles abrasion test (%)	14.3	≤30	T0317-2005
Water absorption (%)	0.32	≤2	T0307-2005
Asphalt adhesion (graduation)	4	≥4	T0616-1993
Impact value (%)	17	≤30	T0322-2000
Firmness (%)	2.9	≤12	T0314-2000
Mud content (%)	0.8	≤1	T0310-2005

**Table 3:** Mineral-filler properties.

Properties	Hydrophilic Coefficient	Water Content (%)	Apparent Density (t/m^3^)	Size Distributions (%)		
				<0.075 mm	<0.15 mm	<0.6 mm
Results	0.634	0.5	2.720	80.7	96.3	100
Criteria	<1	≤1	≥2.50	75~100	90~100	100
Methods	T0353-2000	T0350-1994	T0352-2000	T0351-2000		

**Table 4:** List of design index test results.

Gradation Type	Asphalt Content (%)	Compressive Strength (MPa)			Resilient Modulus (MPa)			Shear Strength (MPa)			Splitting Strength (MPa)		
		90#	30#	RM	90#	30#	RM	90#	30#	RM	90#	30#	RM
Gradation 1	3.9	3.08	6.54	3.74	1293	2581	1173	1.07	1.55	1.52	1.01	1.63	0.87
	4.2	3.23	6.80	3.91	1391	2752	1315	0.99	1.48	1.50	1.19	1.67	0.86
	4.5	2.96	6.73	4.11	1388	2609	1243	0.96	1.33	1.63	1.14	1.71	0.93
	4.8	2.86	6.71	4.39	1235	2407	1162	0.89	1.31	1.45	1.00	1.63	0.96
	5.1	2.75	6.45	3.82	1094	2378	1091	0.85	1.22	1.38	0.94	1.54	0.95
Gradation 2	3.9	2.78	5.89	3.37	1165	2326	1056	1.73	2.46	2.62	0.69	1.64	0.96
	4.2	3.38	7.12	4.09	1456	2880	1376	1.81	2.51	2.66	0.71	1.76	1.04
	4.5	3.02	6.87	4.20	1419	2666	1270	1.98	2.92	2.79	0.79	1.83	1.08
	4.8	3.23	7.59	4.96	1397	2721	1313	1.88	2.67	2.89	0.75	1.77	1.00
	5.1	3.12	7.32	4.33	1243	2701	1238	1.81	2.57	3.07	0.66	1.58	0.97
Gradation 3	3.9	3.58	5.33	3.51	1323	2138	1110	1.67	1.89	2.49	0.89	1.63	1.03
	4.2	3.89	5.86	3.99	1646	2367	1234	2.11	2.06	2.56	1.00	1.74	1.05
	4.5	3.38	5.87	3.78	1528	2104	1326	2.14	2.15	2.61	1.09	1.72	1.10
	4.8	3.26	5.52	3.83	1460	1966	1270	1.96	2.06	2.80	0.98	1.64	1.23
	5.1	3.10	5.07	3.62	1256	2021	1093	1.83	1.94	2.56	0.94	1.57	1.07

**Table 5:** The sensitivity of design indices to asphalt type, gradation type, and asphalt content.

Gradation Type	Asphalt Type	Dynamic Modulus		Compressive Strength		Resilient Modulus		Shear Strength		Splitting Strength	
		*p*	Significance	*p*	Significance	*p*	Significance	*p*	Significance	*p*	Significance
Gradation 1	90#	0.0041	**	0.0523	-	0.1710	-	0.1771	-	0.0204	*
	30#	0.0008	***	0.4557	-	0.0588	-	0.0689	-	0.4392	-
	RM	0.0483	*	0.0492	*	0.1585	-	0.3581	-	0.2447	-
Gradation 2	90#	0.0057	**	0.1127	-	0.1591	-	0.2366	-	0.1109	-
	30#	0.0206	*	0.0733	-	0.1675	-	0.0648	-	0.1105	-
	RM	0.0483	*	0.1516	-	0.0443	*	0.0450	*	0.3659	-
Gradation 3	90#	0.0210	*	0.0325	*	0.1522	-	0.0427	*	0.2240	-
	30#	0.0268	*	0.0991	-	0.0579	-	0.0819	-	0.4635	-
	RM	0.0518	-	0.3828	-	0.3159	-	0.1655	-	0.2329	-

Notes: *** means highly significant; ** means mid-level significance; * means low significance; - means not significant.

**Table 6:** Dynamic modulus values under different control standards. (MPa).

Frequency (Hz)	Permanent Deformation Control Standard (Times/mm)						Relational Expression
	800	2400	2800	3000	4000	5000	
25	4462	10,207	11,643	12,362	15,952	19,543	*DS* = 0.2785 × |E*| − 442
10	2794	7851	9115	9747	12,908	16,068	*DS* = 0.3164 × |E*| − 84
5	2027	6616	7763	8336	11,204	14,072	*DS* = 0.3487 × |E*| + 93
1	682	3890	4692	5093	7098	9103	*DS* = 0.4988 × |E*| + 460
0.5	424	3041	3695	4022	5658	7293	*DS* = 0.6114 × |E*| + 541
0.1	163	1624	1989	2172	3085	3998	*DS* = 1.0953 × |E*| + 621

## Data Availability

The data presented in this study are available on request from the corresponding author.
